# Quantitative analysis of *in vivo* high-resolution microendoscopic images for the detection of neoplastic colorectal polyps

**DOI:** 10.1117/1.JBO.23.11.116003

**Published:** 2018-11-20

**Authors:** Yubo Tang, Alexandros D. Polydorides, Sharmila Anandasabapathy, Rebecca R. Richards-Kortum

**Affiliations:** a Rice University, Department of Bioengineering, Houston, Texas, United States; b Mount Sinai Medical Center, Department of Pathology, New York, United States; c Baylor College of Medicine, Section of Gastroenterology and Hepatology, Houston, Texas, United States

**Keywords:** microendoscope, colorectal cancer, adenomatous polyps, quantitative analysis, computer-aided diagnosis

## Abstract

Colonoscopy is routinely performed for colorectal cancer screening but lacks the capability to accurately characterize precursor lesions and early cancers. High-resolution microendoscopy (HRME) is a low-cost imaging tool to visualize colorectal polyps with subcellular resolution. We present a computer-aided algorithm to evaluate HRME images of colorectal polyps and classify neoplastic from benign lesions. Using histopathology as the gold standard, clinically relevant features based on luminal morphology and texture are quantified to build the classification algorithm. We demonstrate that adenomatous polyps can be identified with a sensitivity and specificity of 100% and 80% using a two-feature linear discriminant model in a pilot test set. The classification algorithm presented here offers an objective framework to detect adenomatous lesions in the colon with high accuracy and can potentially improve real-time assessment of colorectal polyps.

## 1 Introduction

Colorectal cancer (CRC) is the third most common cancer and second leading cause of cancer related deaths in the US.^1^ Adenomatous polyps are precursor lesions with a high risk of progression to CRC; they can be detected and resected during screening colonoscopy. Since colonoscopic polypectomy has been shown to reduce CRC incidence and morbidity,^2^^–^^5^ current guidelines recommend removal of all visible polyps followed by histopathologic diagnosis to determine subsequent surveillance intervals.^6^ The large majority of resected lesions, however, are diminutive (≤5  mm) and small (6 to 9 mm) polyps that rarely harbor advanced histological features and neoplasia.^7^^,^^8^ Therefore, there is increasing interest in developing tools that can accurately characterize polyps *in vivo* to avoid unnecessary polypectomy or histopathological examination following resection, thus reducing the cost of screening colonoscopy.^9^^–^^13^

Routine colonoscopy under white light illumination, however, lacks the capability to differentiate malignant from benign lesions.^14^ To improve the *in vivo* assessment of polyps, advanced imaging technologies have been investigated. Narrow-band imaging and confocal laser endomicroscopy (CLE), for example, have been reported to be highly accurate.^9^^,^^15^^,^^16^ However, these advanced modalities require expensive equipment and operator expertise, and are mostly used in tertiary centers. High-resolution microendoscopy (HRME) is a low-cost imaging tool (<$3500) to resolve subcellular structure in real time. Chang et al.^17^ and Parikh et al.^18^ recently demonstrated the ability of HRME to accurately discriminate neoplastic from nonneoplastic colorectal polyps. Qualitative classification criteria for neoplastic lesions in HRME images were developed by Chang et al.^17^ With appropriate training to interpret HRME images, Parikh et al.^19^ showed that endoscopists could visually assess HRME images to identify neoplastic (adenomatous or cancerous) polyps with accuracy greater than 90%.

Most of the previous studies of advanced imaging technologies rely on subjective interpretation for lesion characterization. In this study, we present a computational algorithm to evaluate HRME images and identify neoplastic polyps. Using histopathology as the gold standard, we developed and evaluated the performance of a computational algorithm to discriminate neoplastic from non-neoplastic colorectal polyps in *in vivo* HRME images. Results show that clinically relevant features can be quantified in a reliable and consistent manner to classify colon HRME images with high accuracy. To facilitate its application for real-time CRC lesion characterization, automated image selection methods can be developed and integrated in the future.

## 2 Methods

### 2.1 Imaging System

The HRME is described in detail elsewhere.^20^ Briefly, the system couples a compact fluorescence microscope with a coherent optical fiber bundle. The 1-mm diameter HRME probe is a coherent fiber bundle consisting of 30,000 individual fibers with a core-to-core spacing of ∼4  μm and a circular field-of-view (FoV) of 720  μm (IGN-08/30, Sumitomo Electric Industries). An LED centered at 455 nm (M455L2, Thorlabs, Newton, New Jersey) is used to provide illumination and a scientific CCD camera (Grasshopper 2, FLIR Integrated Imaging Solutions Inc, Richmond, Canada) is used for fluorescence imaging. Real-time videos are recorded and displayed using a laptop computer at a rate of 10 frames per second.

### 2.2 Patient Enrollment and Imaging Procedure

An *in vivo* clinical trial was conducted in patients undergoing routine screening or surveillance colonoscopy at Mount Sinai Medical Center. The study information was provided to eligible patients and written informed consent was obtained. This study was approved by the IRBs at Mount Sinai Medical Center and Rice University. The clinical trial was registered on Clinicaltrials.gov (registration number NCT01384240).

Colonoscopy was performed by an expert endoscopist (S.A.) using a high-definition white-light endoscope. Polyps identified during white-light examination were further interrogated with the HRME in real time. Prior to HRME imaging, 1 to 4 ml of proflavine (0.01%) was topically applied through a spray catheter (Olympus America, Center Valley, Pennsylvania). The imaged polyps were then removed and submitted for histopathological examination. All biopsies were diagnosed by an expert gastrointestinal pathologist (A.D.P.) using standard criteria.

### 2.3 Quantitative Image Analysis

The original HRME videos and images were reviewed for quality control by reviewers blinded to clinical impressions and histologic diagnosis. First, one reviewer selected frames of high quality from recorded videos. Typically, one or two frames were extracted from each 1-s video clip. Images were discarded if 50% or more of the FoV was not clearly visible (out-of-focus, debris, motion artifact or significant saturation). Second, two independent researchers reviewed the image library containing 200 images from 58 sites to select a single representative image per site with the best image quality. When heterogeneous candidate images of one site passed the initial quality control, previously established qualitative criteria by Chang et al.^17^ were applied to select a single image presenting the most severe imaging features.

**Fig. 1:**
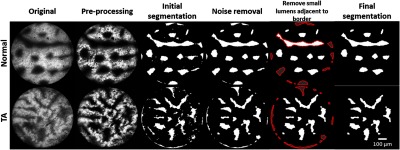
Lumen segmentation algorithm in two representative images (one diagnosed as normal and one as tubular adenoma). Before segmentation, the fiber patterns were removed, and image contrast was enhanced. The images were then converted into a binary image and small structures arising from noise were removed. The next step consisted of identifying lumens on the image border (outlined in red) and removing these partial lumens if they contained only a small fraction of the total area (gray lumens outlined in red). Partial lumens on the border containing a significant luminal area (white lumens outlined in red) were included in the final segmentation image. TA: tubular adenoma.

A variety of quantitative features were calculated to evaluate their potential diagnostic ability (Table 1). Luminal morphology (e.g., lumen caliber, shape, and size) has been shown to be critical in the qualitative differentiation of neoplastic from benign polyps.^17^ To quantitatively evaluate these metrics, we developed an automated algorithm to segment lumens in HRME images, as illustrated in Fig. 1. First, images were preprocessed to remove the fiber bundle patterns with a low-pass filter and enhance the overall contrast. An initial segmentation was performed using a histogram-based threshold. Segmentation noise was then removed through morphologic processing including image opening and closing. In the next step, we identified segmented regions that fell on the borders of the images and outlined them in red. Since these outlined regions may either arise from noise along the borders or represent only a small fraction of imaged lumens, regions smaller than 1% of the image size were marked in gray and excluded from the analysis. Using the final segmentation results, we calculated the eccentricity, diameter (as the equivalent diameter of a circle with the same area), and perimeter for each of the segmented lumens. Their mean values, coefficient of variation (CV), skewness, and kurtosis on each image were used as separate features.

**Table 1:** Description of features calculated for each colon HRME image.

Metric	No. of features	Description
Lumen segmentation	12	Mean, coefficient of variation, skewness, and kurtosis of lumen eccentricity, diameter, perimeter
Frequency content	10	Frequency distribution of pixel values in each of the 10 partitions of the power spectrum

It has also been shown that texture features can be used to extract important spatial information about glandular architecture.^21^^–^^25^ Since the colon mucosa images show quasiperiodic 2-D luminal patterns and texture features, frequency analysis was performance in the Fourier frequency domain. A power spectrum was calculated after a 2-D Fourier transform and evenly divided into 10 partitions using a previously reported method.^21^ Specifically, a maximum square region within the FoV is used for frequency content analysis. Its discrete Fourier transform is divided into 10 partitions, with the first partition representing the lowest spatial frequency range and the 10th representing the highest. The frequency contents are summed up in each partition and their relative contributions to the total power spectral density are calculated, resulting in 10 distinct features that represent low to high spatial frequency ranges.

### 2.4 Algorithm Development

The entire dataset was divided into a training/validation set and a test set. In the training/validation set that contains the first 40 images, we performed 10-fold cross-validation and selected the number of features to build a predictive model. The model was then evaluated using a separate test set that contains the remaining 18 images.

In the 40-image training/validation set, calculated features were assessed in the subsequent discriminant analysis to classify neoplastic (tubular adenoma, tubulovillous adenoma and sessile serrated adenoma, cancer) from non-neoplastic (normal mucosa, inflammatory polyp, hyperplastic polyp) polyps. First, the classification potential of each feature was evaluated with a Student’s t-test in a univariate analysis. The mean values of a single input feature in two groups were compared and its significance level was determined.

We also performed a multivariate analysis to build a two-class classifier based on a subset of up to five features. The number of features to construct the final predictive model was determined through a 10-fold cross-validation in the 40-image set. In each fold of the cross-validation, a 90%/10% split was used to separate the training set (36 images) and the validation set (4 images). First, a best performing k-feature model was selected to maximize the classification area under the curve (AUC) in the training set. Specifically, k features were used to compute the posterior probability through a linear discriminant analysis. A receiver operating characteristic (ROC) curve was constructed with histopathology as the gold standard and AUC was maximized to select a best performing model. Second, the model was validated in the validation set to calculate the classification error. For models that contain k features, the overall performance was evaluated using the cross-validation error, defined as the average classification error in 10 folds. The number of features in the final predictive model was determined to limit the cross-validation error with a small set of features. As the number of features was decided, the final predictive model was built in the full training/validation set and its performance in the separate test set was reported.

## 3 Results

### 3.1 Patient Enrollment: Sites and Images

We extracted images from original videos for 70 sites in 52 patients with corresponding histopathology. Images for 12 sites failed to pass the quality control and the reviewers reached consensus to select a single image per site for the remaining 58 sites in 46 patients. In this 58-image set, 36 sites are diagnosed as non-neoplastic (normal mucosa, inflammatory polyp, hyperplastic polyp) and 22 are diagnosed as neoplastic (tubular adenoma, tubulovillous adenoma, sessile serrated adenoma and cancer). The detailed histopathologic diagnosis is shown in Table 2.

**Table 2:** Site information based on histopathology.

Category	Histopathology Dx	No. of sites
Non-neoplastic	Normal mucosa	10
	Hyperplastic polyp	20
	Inflammatory polyp	6
Neoplastic	Tubular adenoma	16
	Tubulovillous adenoma	4
	Serrated adenoma	2
Total		58 (46 pts)

**Fig. 2:**
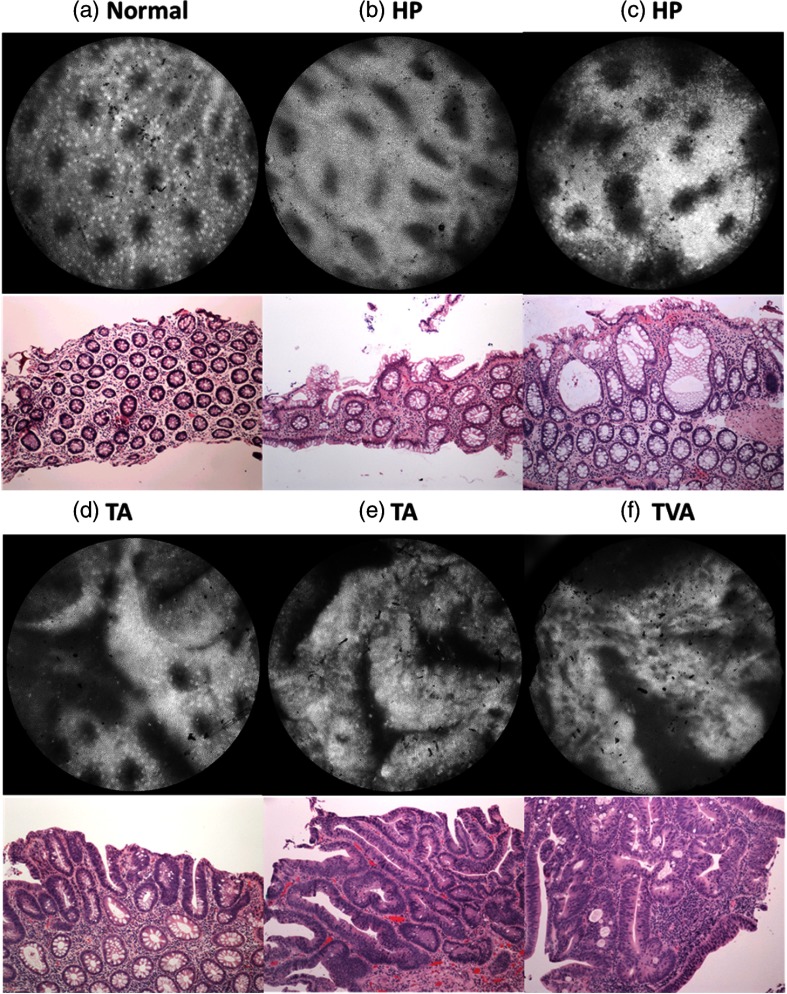
Representative *in vivo* images and the corresponding pathology. (a) The normal colorectal mucosa reveals uniform glandular distribution across the image. (b) The luminal size and shape are slightly varied with occasional widening in the hyperplastic polyp. (e, f) In contrast, both TA and TVA images show linear crypts; villous structures are observed in TVA. HRME images showing mixed morphology are also shown. (c) image is obtained from a hyperplastic site, which shows widened and sometimes connected lumens. (d) small round lumens, as well as wide and linear openings, are observed, and the site is diagnosed as TA. HP, hyperplastic polyp; TA, tubular adenoma; TVA, tubulovillous adenoma.

Figure 2 shows representative images for sites diagnosed as normal mucosa (a), hyperplastic polyp (b, c), tubular adenoma (d, e), and tubulovillous adenoma (f) with the corresponding histopathology. Normal colon mucosa in Fig. 2(a) is characterized by the uniform glandular distribution across the entire image; round or oval openings are present with similar shapes and sizes. The lumens become slightly distorted in the hyperplastic polyp in Fig. 2(b); while some lumens appear elongated, they are confined by intact glandular borders. Both tubular adenoma and tubulovillous adenoma in Figs. 2(e) and 2(f) show larger and more linear lumens. In addition to images showing distinct benign or neoplastic features, images that reveal mixed morphology are also shown in Figs. 2(c) and 2(d). In Fig. 2(c), the lumens are more widened than Fig. 2(b) and can appear connected. In the tubular adenoma in Fig. 2(d), wide and linear openings, as well as small round lumens are observed, suggesting structural transitions between benign and neoplastic lesions. All these structural features are confirmed in the corresponding histopathology images.

### 3.2 Algorithm Development: Model Selection and Performance

Table 3 lists features that show statistically significant differences between the neoplastic and non-neoplastic groups in the training/validation set (p<0.05). The features are shown in descending order based on the p-value. Differences in the mean values are statistically significant for 6 of the 22 features.

**Table 3:** Features that show statistically significant differences between neoplastic and non-neoplastic groups (p<0.05).

Feature	p value
Mean of luminal perimeter	0.001
Frequency content 7	0.004
Mean of luminal diameter	0.009
Variance of luminal diameter	0.013
Frequency content 4	0.016
Variance of luminal perimeter	0.024

**Fig. 3:**
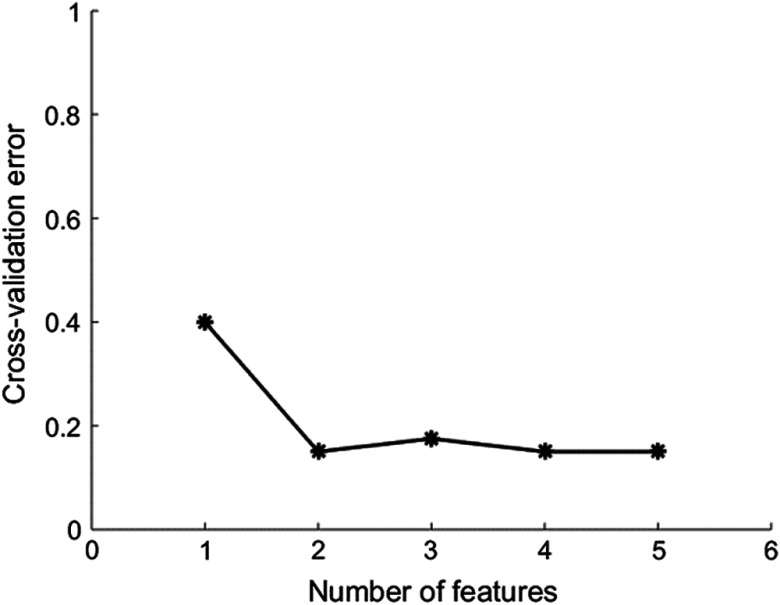
Cross-validation error for k-feature models. The linear classifiers evaluated in the plot comprise one to five features. As the number of features increased, the cross-validation error began to plateau when more than two features were used. As a result, a two-feature model was selected to build the final predictive model.

In the multivariant analysis, we monitored the performance of k-feature models using cross-validation error as added features were selected from the 22-feature set. As shown in Fig. 3, the cross-validation error began to plateau when more than 2 features were used. As a result, two features were used to build the final predictive model.

**Fig. 4:**
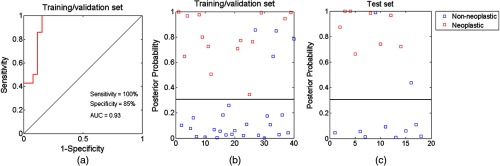
Performance of the linear discriminant model based on two features. (a)–(c) The final predictive model was trained in the training/validation set, resulting in an AUC of 0.93, and a sensitivity and specificity of 100% and 85% at the Q-point. The model with a fixed posterior probability threshold was then evaluated in the test set, achieving a sensitivity and specificity of 100% and 80%, respectively.

The final predictive model was constructed to maximize the AUC with two features in the training/validation set, and then evaluated in the test set. Selected features were frequency content 7 and mean of luminal perimeter. The ROC curve in the training/validation set shows an AUC of 0.93 for classification of neoplastic from non-neoplastic polyps; a posterior probability threshold was chosen at the Q-point, resulting in a sensitivity and specificity of 100% and 85%. The two-feature linear discriminant model was then tested in the test set, achieving a sensitivity and specificity of 100% and 80%, respectively. Scatter plots of the posterior probability in the two datasets are also shown in Fig. 4.

## 4 Discussion

In this research paper, we report a computer-aided algorithm to differentiate benign from adenomatous polyps using high-resolution microendoscopic images. Using a two-feature linear discriminant model, we demonstrate that adenomatous polyps can be classified with a sensitivity and specificity of 100% and 85% in the training/validation set, and a sensitivity and specificity of 100% and 80% in a separate test set. Both selected features in the final model are statistically different between the non-neoplastic and neoplastic groups in the training/validation set. The first feature is frequency content in the seventh partition of the power spectrum. This partition belongs to the high frequency range and its relative contribution is significantly lower in the neoplastic images, which can be attributed to the loss of fine structures and widening of more homogenous regions, such as lumens in these lesions. The other feature is the mean of luminal perimeter, which is significantly higher in the neoplastic group. This is consistent with the previous qualitative observation that larger openings are associated with adenomatous lesions. While promising, results presented here should be further confirmed in a larger sample size. To further evaluate and optimize the classifier in real time, fully automated image acquisition and analysis can be also enabled by integrating an objective frame selection algorithm.^26^ When coupled with frame selection methods optimized for columnar epithelium, the classification algorithm presented here has the potential to assist in clinical decision-making at the point of care.

Qualitative microendoscopic criteria for visual inspection have been developed to diagnose neoplastic polyps using CLE and HRME, and their performance and interobserver variability have been studied. In endoscope-based confocal laser endoscopy (eCLE), a substantial interobserver agreement has been reported and the accuracy of three observers ranged from 85.6% to 95.6%.^27^ In probe-based confocal laser endoscopy (pCLE), a moderate interobserver agreement (k=0.55) was reported with a sensitivity and specificity of 76% and 72% for three users by Gómez et al.;^28^ similarly, Kuiper et al.^29^ found a moderate interobserver agreement with a sensitivity and specificity of 66% and 83% for five observers. As an inexpensive alternative to the CLE, HRME demonstrated a sensitivity and specificity of 70% and 94% with a substantial agreement in a previous qualitative study.^17^ The computational algorithm described here presents a quantitative framework to classify images with high accuracy. Future work is necessary to integrate automated frame selection for its application in the clinical practice.

Quantitative and automated analysis of CLE images have also been developed in a range of clinical applications, such as cancer detection in the brain, oral cavity, and colon; both conventional classifiers and deep learning approaches have been expolored.^30^^–^^34^ Unlike intravenous fluorescein used in CLE that diffuses nonspecifically, proflavine preferentially stains nuclei and thus highlights nuclear morphometry and glandular patterns.^35^ In addition, HRME offers a low-cost alternative (<$3500) to CLE and has the potential for cancer screening in low-resource and community settings. Like other high-resolution modalities, one limitation of the imaging approach in this research is the small FoV of the HRME, which is inherently limited by the diameter of the probe (720  μm). As shown in Fig. 1, some lumens can only be partially imaged in a single frame. The segmentation algorithm was optimized to exclude less significant fractions of lumens, which may skew the calculation of morphological parameters. In the future, the FoV and thus the sampling size can be potentially expanded with a mosaicking algorithm.^36^

The classification algorithm presented here provides clinicians an objective and reliable framework to characterize colorectal polyps with high accuracy. It can be particularly beneficial in low-resource settings, where comprehensive training in new imaging techniques may not be adequately provided, and thus facilitates the dissemination of HRME as a cost-effective imaging tool. Prospective studies are warranted to evaluate and optimize its performance to improve real-time assessment of colon polyps.
